# Enhanced recovery pathways improve postoperative outcomes in haemorrhoid surgery: a narrative review

**DOI:** 10.3389/fsurg.2026.1728394

**Published:** 2026-02-04

**Authors:** Alin Kraft, Cosmin Alec Moldovan, Elena Rusu, Daniel Cochior

**Affiliations:** 1 Department of Medical-Surgical and Prophylactic Disciplines, Faculty of Medicine, Titu Maiorescu University, Bucharest, Romania; 2 Department of General Surgery, “General Doctor Aviator Victor Anastasiu” National Aeronautical and Space Medicine Institute, Bucharest, Romania; 3 Department of General Surgery, Witting Clinical Hospital, Bucharest, Romania; 4 Department of General Surgery, Sanador Hospital, Bucharest, Romania

**Keywords:** haemorrhoidectomy, enhanced recovery after surgery, perioperative care protocols, proctologic surgery, surgical pathway standardization

## Abstract

Haemorrhoid surgery, while typically performed as a low-risk outpatient procedure, is frequently associated with considerable postoperative discomfort, delayed return to daily activities, and occasional unplanned healthcare visits. Enhanced recovery protocols, originally developed to improve outcomes in major abdominal surgery, offer a structured, evidence-based framework for optimizing perioperative care and accelerating recovery. This review explores whether these principles can be effectively applied to the context of haemorrhoidectomy. Key components such as patient education, multimodal pain control, minimal preoperative fasting, early mobilization, and structured discharge planning are conceptually well-suited to the needs of patients undergoing benign anorectal surgery. Early clinical experiences and extrapolated data from related surgical settings suggest that even minor procedures may benefit from standardized recovery pathways, with reported reductions in postoperative pain scores of up to forty percent, faster return to function by two to three days, and nearly fifty percent fewer unplanned return visits. These findings support the potential for improved pain management, reduced variability in care, and enhanced patient satisfaction. However, current evidence remains limited, with few studies specifically addressing the impact of enhanced recovery protocols in haemorrhoidectomy. This article synthesizes the available literature, highlights potential advantages and implementation challenges, and advocates for the development of a simplified, procedure-specific perioperative pathway tailored to the characteristics of this common surgical intervention. The integration of digital follow-up tools and individualized care strategies may further support recovery and reduce complications in this setting. Overall, a targeted and pragmatic approach to enhanced recovery in haemorrhoid surgery represents a clinically meaningful opportunity to improve outcomes and modernize care delivery in proctology.

## 1 Introduction

Haemorrhoidal disease remains one of the most prevalent benign anorectal conditions worldwide, with an estimated incidence affecting up to 4% of the general population and a substantial proportion requiring surgical intervention (1). While conventional haemorrhoidectomy techniques, such as the Milligan-Morgan or Ferguson procedures, have demonstrated long-term efficacy, they are frequently associated with significant postoperative pain, prolonged recovery, and variable patient satisfaction. These challenges have prompted the exploration of structured perioperative strategies aimed at mitigating surgical stress and enhancing functional recovery.

Enhanced Recovery After Surgery (ERAS) protocols represent a multimodal, evidence-based approach designed to optimize perioperative care, reduce postoperative complications, and expedite convalescence (2, 3). Initially developed for colorectal resections, ERAS pathways have been validated across a wide range of surgical domains, including urology, gynecology, hepatopancreatobiliary surgery, and increasingly in proctologic procedures (4, 5). Core components of ERAS include preoperative patient education, minimization of fasting, early enteral nutrition, multimodal analgesia with opioid-sparing techniques, early mobilization, and structured discharge planning (6).

In the context of major colorectal surgery, the implementation of ERAS protocols has consistently yielded improvements in postoperative outcomes, including shorter hospital stays, lower complication rates, faster return of bowel function, and reduced healthcare costs (7, 8). More recently, these principles have been extrapolated to minor anorectal operations performed in outpatient settings, including haemorrhoidectomy. Emerging evidence suggests that selected ERAS components may be equally applicable and beneficial in the management of benign proctologic conditions, where the burden of postoperative discomfort, delayed return to activity, and unplanned readmissions remains clinically relevant (9–11).

However, the direct application of ERAS principles to haemorrhoid surgery remains insufficiently documented, with limited high-quality studies focused specifically on this subset of patients. Most available data originate from broader cohorts involving ambulatory anorectal surgeries or retrospective analyses of enhanced recovery protocols tailored to colorectal practice (12, 13). In addition, the adaptation of ERAS to low-risk, high-volume outpatient procedures raises unique questions regarding feasibility, adherence, and cost-effectiveness.

This descriptive review aims to systematically examine the existing literature regarding the role of ERAS protocols in haemorrhoid surgery, highlighting potential benefits, areas of uncertainty, and future directions for evidence-based protocol development in benign proctology.

Although the initial implementation of ERAS protocols targeted major abdominal procedures, their progressive adaptation across surgical subspecialties—including gynecologic, urologic, and orthopedic surgery—has revealed fundamental perioperative principles of broad applicability. These multidisciplinary insights support the rationale for evaluating ERAS integration in less invasive, high-volume outpatient procedures such as haemorrhoidectomy.

To support this review, a structured literature search was conducted in PubMed, Scopus, Web of Science, and Google Scholar, covering the period from 2010 to 2025. Keywords included “enhanced recovery,” “ERAS,” “haemorrhoidectomy,” “proctologic surgery,” “minor anorectal surgery,” and “ambulatory colorectal surgery.” Studies were selected based on their relevance to ERAS implementation in benign anorectal surgery, with emphasis on perioperative outcomes, feasibility, recovery protocols, and patient-centered metrics. Systematic reviews, clinical guidelines, and both prospective and retrospective studies were included.

## 2 Rationale for ERAS in minor proctologic surgery

Although haemorrhoidectomy is typically regarded as a minor outpatient procedure, it is frequently associated with considerable postoperative morbidity. Pain, urinary retention, anxiety, and delayed return to daily activities are common complications, even in otherwise healthy individuals. These issues, although rarely life-threatening, can significantly affect quality of life, increase healthcare costs, and reduce patient satisfaction. Pain in particular has been consistently identified as the most troublesome postoperative symptom, often leading to prolonged analgesic use and delayed functional recovery (14, 15).

Enhanced Recovery After Surgery (ERAS) protocols—first validated in elective colorectal resections—have proven effective in mitigating surgical stress, reducing complications, and accelerating return to function through multimodal, evidence-based perioperative care strategies (11, 16). Their success in colorectal and other surgical disciplines has encouraged efforts to adapt ERAS principles to minor proctologic procedures, including haemorrhoidectomy. Recent studies show that structured pathways incorporating patient education, opioid-sparing multimodal analgesia, early mobilization, and standardized discharge planning can improve postoperative outcomes even in ambulatory anorectal surgery (2, 5).

The rationale for this adaptation is supported by the demographic profile of patients undergoing haemorrhoid surgery—typically young, working-age individuals who require a fast and predictable return to normal activity. For this group, the benefits of ERAS protocols are highly relevant, especially when elements such as minimal fasting, early postoperative feeding, and targeted anxiolysis are integrated. A prospective ERAS pathway developed specifically for outpatient anorectal surgery demonstrated significantly lower pain scores, reduced opioid consumption, and a marked decrease in unplanned healthcare contacts (1).

The rationale for applying ERAS principles to haemorrhoidectomy lies in their ability to address specific recovery barriers—pain, delayed mobilization, patient anxiety, and inconsistent postoperative instructions. The main justifications for ERAS implementation in haemorrhoid surgery are summarized in Table 1.

**Table 1:** Key messages Supporting ERAS Implementation in haemorrhoid surgery.

Challenge/Characteristic	Implication in Haemorrhoid Surgery	ERAS Rationale/Benefit
Postoperative pain	Most common complication; reduces mobility and delays recovery	Multimodal, opioid-sparing analgesia improves comfort and function
Unplanned readmissions or consultations	Frequently triggered by unmanaged symptoms or lack of education	Structured counseling and discharge planning reduce post-discharge complications
Young, working-age patient population	High expectations for rapid return to work and function	Streamlined recovery protocols meet patient-centered goals
Outpatient setting with early discharge	Limits opportunities for extended monitoring or correction of complications	Digital follow-up tools and structured transitions of care can maintain continuity
High variability in perioperative care practices	Inconsistent outcomes, non-standardized pain management	ERAS standardization reduces variability and supports predictable recovery
Minimal systemic insult compared to major surgery	Misleading perception that structured recovery is unnecessary	Even “minor” surgery benefits from optimized perioperative care
Lack of nutritional or bowel prep requirements	Simpler preoperative setup	Allows for focused ERAS bundle: education, analgesia, feeding, early ambulation
High procedural volume across centers	Large-scale impact potential	Scalable interventions enhance efficiency and quality at system level

Conceptual rationale for the application of Enhanced Recovery After Surgery (ERAS) principles in haemorrhoidectomy. The table outlines key perioperative challenges specific to minor proctologic surgery, their implications for patient outcomes, and the potential role of ERAS interventions in mitigating these risks.

Moreover, evidence from broader ERAS implementation in benign anal and fistula surgery supports its safety and feasibility in low-risk, high-volume proctologic procedures (17). When combined with digital follow-up platforms and real-time symptom monitoring, enhanced recovery pathways may also help address long-standing gaps in continuity of care following same-day discharge. Such interventions not only improve clinical outcomes, but also reduce variability in care delivery and optimize resource utilization (10).

While not all classical ERAS elements are required in minor surgery, a focused adaptation—emphasizing education, analgesia, minimal perioperative fasting, and structured follow-up—could modernize the perioperative management of haemorrhoidectomy and improve the patient experience in a scalable, cost-effective manner. Given the rising emphasis on outpatient surgery and value-based care, haemorrhoidectomy presents an ideal platform for expanded implementation of enhanced recovery protocols.

## 3 Key ERAS elements adapted for haemorrhoid surgery

The concept of Enhanced Recovery After Surgery (ERAS) is built upon the coordinated implementation of multimodal, evidence-based interventions that aim to reduce the physiological and psychological stress of surgery, facilitate earlier recovery, and improve overall outcomes. While originally developed for major abdominal operations, growing evidence supports the feasibility and effectiveness of ERAS principles in less invasive, high-frequency procedures such as haemorrhoidectomy. Recent advances in perioperative optimization and ambulatory proctologic care have highlighted the opportunity to apply selected ERAS elements to this setting in a pragmatic and resource-conscious manner.

While originally developed for major abdominal operations, growing evidence supports the feasibility and effectiveness of ERAS principles in less invasive, high-frequency procedures such as haemorrhoidectomy. Recent advances in perioperative optimization and ambulatory proctologic care have highlighted the opportunity to apply selected ERAS elements to this setting in a pragmatic and resource-conscious manner. These core components and their adaptations to haemorrhoid surgery are summarized in Table 2.

**Table 2:** Key ERAS elements adapted for haemorrhoid surgery.

ERAS Component	Adaptation for Haemorrhoid Surgery	Clinical Benefit
Preoperative Education	Targeted counseling on pain expectations, wound care, and recovery milestones	Reduces anxiety, improves satisfaction, and lowers unplanned visits
Minimized Fasting & Carbohydrate Loading	Clear fluids up to 2 h pre-op; optional carb drinks for general anesthesia cases	Reduces insulin resistance, discomfort, and perioperative stress
Anesthetic Strategy	Local or regional anesthesia with light sedation preferred over general anesthesia	Minimizes nausea, urinary retention, and facilitates same-day discharge
Multimodal Analgesia	NSAIDs, acetaminophen, and local anesthetic infiltration (e.g., ropivacaine)	Reduces opioid use and associated side effects (constipation, sedation)
Early Oral Intake	Resume fluids and soft foods shortly after recovery from anesthesia	Promotes gut motility, prevents constipation, enhances comfort
Early Mobilization	Encourage walking same day of surgery and return to daily activities within 2–5 days	Improves circulation, bowel function, and psychological recovery
Structured Discharge & Follow-up	Clear criteria and digital/telemedicine-based monitoring where feasible	Improves continuity of care and reduces avoidable readmissions
Avoidance of Unnecessary Interventions	No routine catheters, anal packing, or antibiotics unless clinically indicated	Reduces iatrogenic complications and enhances patient comfort

Adaptation of core ERAS components for haemorrhoidectomy. This table summarizes how selected ERAS elements—originally designed for major inpatient surgery—can be modified for use in outpatient proctologic procedures, highlighting specific clinical applications and anticipated benefits.

### 3.1 Preoperative education and patient counseling

Patient education is a cornerstone of ERAS protocols. Structured preoperative counseling has been shown to reduce anxiety, clarify expectations, and significantly improve adherence to postoperative instructions (1, 2). In the context of haemorrhoid surgery—often burdened by patient misconceptions, fear of pain, and uncertainty regarding outcomes—preoperative information regarding the procedure, anesthesia type, anticipated pain, dietary management, and wound care is essential. Studies have confirmed that patients who receive targeted education experience fewer unplanned contacts with healthcare providers and report greater satisfaction (18).

### 3.2 Minimization of preoperative fasting and carbohydrate loading

Classic preoperative fasting policies are being replaced in ERAS guidelines by more physiological regimens. Patients undergoing anorectal procedures can safely consume clear fluids up to two hours before anesthesia and benefit from preoperative carbohydrate loading, which reduces insulin resistance, catabolism, and subjective hunger (3). Although many haemorrhoidectomies are performed under spinal or local anesthesia, these recommendations may still be beneficial, particularly in sedated or anxious patients. Guidelines also suggest reducing fasting for solids to 6 h, which is well-tolerated and safe across surgical populations (4).

### 3.3 Anesthetic strategy: local, regional, or light general anesthesia

The choice of anesthetic technique significantly influences recovery. ERAS promotes the use of anesthetic approaches that minimize systemic impact, facilitate early discharge, and reduce postoperative nausea and sedation. In haemorrhoidectomy, regional anesthesia (caudal or spinal blocks), local infiltration, or light sedation are preferable over general anesthesia, especially in ambulatory settings (5). A recent study by Tomasicchio et al. demonstrated that local or saddle block anesthesia in a tailored ambulatory protocol yielded low complication rates, minimal postoperative pain, and rapid return to function (19).

### 3.4 Multimodal, opioid-sparing analgesia

Postoperative pain is consistently reported as the main limitation to rapid recovery following haemorrhoidectomy. ERAS encourages a multimodal analgesic strategy combining non-opioid agents such as NSAIDs and acetaminophen with local anesthetic techniques, including perianal blocks or pudendal nerve infiltration. Xu et al. showed that long-acting analgesia protocols using intraoperative infiltration of ropivacaine significantly reduced pain scores and need for rescue opioids (20). The PROSPECT guideline further supports the use of regional blocks and avoidance of routine opioids in anorectal surgery, citing reduced nausea, urinary retention, and sedation (18).

### 3.5 Early oral intake and avoidance of routine nasogastric tubes

Although nasogastric decompression is largely irrelevant in minor proctologic surgery, the principle of early refeeding remains essential. Encouraging oral intake within hours after recovery from anesthesia helps maintain gut motility, prevents constipation—a major concern in anorectal procedures—and contributes to improved wellbeing (21). Data from colorectal ERAS pathways show that early refeeding is safe, even in emergency contexts, and should be applied unless specific contraindications exist (22).

### 3.6 Early mobilization and return to activity

ERAS emphasizes early ambulation to prevent complications such as urinary retention, ileus, and thromboembolism. While these risks are lower in ambulatory haemorrhoid surgery, mobilization supports better circulation, improved bowel function, and patient autonomy. Patients are encouraged to resume light activities on the day of surgery and return to normal function within 2–5 days. Studies show that this approach is safe and associated with faster recovery and lower complication rates, particularly when pain is adequately controlled (13, 16).

### 3.7 Clear discharge criteria and structured outpatient follow-up

Well-defined discharge criteria are essential for safe same-day discharge. These include stable vital signs, ability to ambulate, void spontaneously, and tolerate fluids. ERAS protocols promote written discharge instructions and structured outpatient follow-up. Digital health tools—such as mobile apps and automated symptom tracking platforms—can facilitate postoperative monitoring, improve communication, and prevent unnecessary readmissions (10, 23). A systematic review by Gani et al. demonstrated that mobile health interventions improve compliance with ERAS pathways and patient engagement in surgical recovery (9).

### 3.8 Avoidance of unnecessary interventions

Another hallmark of ERAS is reducing interventions that do not improve outcomes or may even hinder recovery. In haemorrhoidectomy, this includes avoidance of urinary catheterization unless clinically indicated, selective use of prophylactic antibiotics, and the elimination of routine anal packing or long-term dressings. These strategies reduce pain, lower infection risk, and improve comfort without compromising safety (11, 12).

## 4 Evidence from outpatient anorectal and benign colorectal surgery

While ERAS protocols have been extensively validated in major colorectal resections, their formal application to minor anorectal and benign colorectal procedures remains less defined. Nevertheless, a growing body of literature supports the feasibility and clinical benefit of selectively adapting ERAS principles to ambulatory proctologic surgery, especially in procedures such as haemorrhoidectomy, fistulotomy, and anal fissure repair.

Recent studies evaluating ERAS implementation in minor anorectal and benign colorectal procedures support its feasibility and potential benefits in outpatient proctologic care. A summary of key studies in this domain is presented in Table 3.

**Table 3:** Summary of Key studies on ERAS in ambulatory anorectal and benign colorectal surgery.

Author (Year)	Study Type	Population/Procedure	ERAS Components	Main Findings
Parrish et al. (2018) (1)	Retrospective cohort study	187 ambulatory anorectal procedures (hemorrhoids, fissures, fistulas)	Education, opioid minimization, structured discharge, early mobilization	↓ Post-op pain, ↓ unplanned visits by 50%, no ↑ in complications
Catarci et al. (2020) (2)	Prospective study	130 outpatient proctologic surgeries	Early feeding, early ambulation, minimal dressing, pain control	High adherence, low pain, no ↑ complications or readmissions
Yao et al. (2024) (24)	Randomized controlled trial	124 patients with anorectal surgery (hemorrhoidectomy, sphincterotomy)	Multimodal analgesia, early mobilization, standardized pathway	↓ Opioid use, ↑ satisfaction, faster return to activity
Chemali et al. (2016) (5)	Systematic review (9 studies)	Various minor proctologic procedures	Varied (pain control, mobilization, feeding)	Consistent ↓ pain, ↓ hospital stay, improved satisfaction
Talboom et al. (2024) (25)	Observational cohort study	Ambulatory anorectal surgeries	—	Nearly 50% sought unplanned care post-op; need for structured ERAS
Lohsiriwat & Jitmungngan (2022) (8)	Systematic review	Post-hemorrhoidectomy pain management	Multimodal analgesia, no packing, early mobility	↓ Pain, better recovery, aligned with ERAS principles

Summary of major published studies evaluating the use of Enhanced Recovery After Surgery (ERAS) protocols in ambulatory anorectal and benign colorectal procedures. The table highlights study type, ERAS components applied, and key clinical outcomes reported.

A landmark single-center retrospective study by Parrish et al. remains one of the earliest and most influential contributions in this area. The authors implemented an ERAS pathway for 187 patients undergoing same-day anorectal procedures, including haemorrhoids, fissures, and fistulas. Their protocol emphasized preoperative counseling, standardized anesthesia, opioid minimization, early mobilization, and structured discharge instructions. Compared to a historical cohort, patients in the ERAS group had significantly reduced pain scores and a 50% reduction in unplanned returns to care within 30 days—without any increase in complications—providing compelling evidence of clinical benefit even in minor interventions (1).

Expanding on this, a prospective trial by Catarci et al. evaluated 130 patients undergoing ambulatory proctologic procedures under a simplified ERAS protocol. Their protocol included immediate postoperative oral intake, early ambulation, minimal use of dressings, and structured pain management. The study reported high adherence, minimal complications, and rapid return to function, confirming the practicality and safety of ERAS in proctology beyond colorectal oncologic surgery (2).

More recently, a randomized trial by Yao et al. (24) tested a multimodal ERAS pathway in 124 patients undergoing ambulatory anorectal surgery, including haemorrhoidectomy and lateral sphincterotomy. The intervention group showed significantly lower postoperative opioid use, earlier ambulation, and greater satisfaction scores at postoperative day 7. The study adds high-quality evidence to the field and confirms that ERAS adaptations can be rigorously tested and quantified in same-day proctologic procedures (24).

In a complementary systematic review by Chemali et al., nine studies were analyzed regarding ERAS protocols in minor proctologic surgery. Despite some heterogeneity in design and protocol definitions, the review consistently highlighted reductions in postoperative pain, hospital stay, and analgesic requirements. The authors concluded that while more randomized studies are needed, the preliminary data strongly support ERAS feasibility in this context (5).

Further indirect support is provided by experiences in benign colorectal resections, including diverticulitis and non-neoplastic rectal procedures. Studies applying ERAS in these cases have consistently demonstrated improved postoperative outcomes, particularly when the protocols are simplified and adapted to disease severity. These findings suggest that even though benign colorectal resections are more invasive than anorectal procedures, many of the same recovery principles apply—especially early feeding, mobilization, and opioid-sparing analgesia (3, 4).

Importantly, data from observational cohorts, such as the study by Talboom et al., reveal that unplanned healthcare utilization after ambulatory anorectal surgery remains surprisingly high, with nearly half of patients seeking postoperative assistance. The most common reasons include pain, bleeding, and uncertainty about recovery trajectory. These findings underscore the urgent need for structured perioperative pathways—like ERAS—that provide better education, pain control, and discharge planning (25).

Finally, a 2022 systematic review by Lohsiriwat and Jitmungngan focused exclusively on strategies to reduce post-hemorrhoidectomy pain. It found that multimodal analgesia, avoidance of packing, and early mobility significantly impact recovery trajectories. Their recommendations are directly aligned with key ERAS principles and further support their relevance in minor anorectal procedures (8).

Despite these encouraging results, most studies to date are limited by moderate sample sizes, retrospective designs, and variability in protocol components. The lack of large-scale, procedure-specific randomized controlled trials remains a barrier to universal ERAS adoption in haemorrhoidectomy. Nonetheless, the accumulating evidence—across retrospective, prospective, and randomized studies—indicates that thoughtfully adapted ERAS pathways can improve clinical outcomes without increasing risk, and merit further formal validation in this high-volume, functionally impactful surgical domain.

## 5 Lessons from ERAS implementation in other surgical fields

The Enhanced Recovery After Surgery (ERAS) paradigm has been successfully implemented across a wide array of surgical specialties, beyond its original application in colorectal surgery. Experiences drawn from upper gastrointestinal, hepatobiliary, gynecologic, urologic, orthopedic, and even emergency surgical fields provide valuable insights into which ERAS components offer universal benefits, which require context-specific adaptation, and which may not be appropriate in minor outpatient procedures such as haemorrhoidectomy.

In major abdominal surgery, particularly colorectal and pancreatic resections, ERAS has consistently demonstrated reductions in complication rates, length of hospital stay, and healthcare costs, while improving return to baseline function and patient satisfaction (3, 11). These programs typically incorporate preoperative carbohydrate loading, early mobilization, opioid-sparing analgesia, early oral intake, and the elimination of drains and catheters. Similarly, gynecologic and urologic ERAS pathways have validated the benefits of structured counseling, regional anesthesia, and multimodal pain management, often in minimally invasive or laparoscopic settings (26, 27).

However, when translating ERAS to minor outpatient procedures, such as haemorrhoidectomy, several components require reevaluation. For example, the role of preoperative carbohydrate loading or thromboembolism prophylaxis may be limited in healthy, low-risk patients. In orthopedic and day-case breast surgery, simplified ERAS models focusing on key interventions—such as preemptive analgesia, patient education, and structured discharge planning—have proven both feasible and effective (28, 29). These streamlined protocols provide a useful framework for adapting ERAS to ambulatory proctologic interventions.

Furthermore, evidence from emergency colorectal surgery shows that even in unplanned, high-risk contexts, adherence to ERAS principles—especially early feeding, minimal fluid overload, and multimodal analgesia—can improve outcomes without increasing complications (22). These findings reinforce the notion that ERAS is not inherently limited to complex elective procedures but can be pragmatically adapted to more variable or resource-limited settings.

Nonetheless, caution is warranted. The heterogeneity of ERAS implementation across specialties has highlighted several challenges, including inconsistent adherence, over-standardization, and a lack of flexibility to accommodate individual patient needs. A critical review by Ljungqvist et al. emphasized that the success of ERAS depends not only on the selection of components but also on institutional culture, team coordination, and patient engagement (30). These factors become especially relevant in outpatient settings, where perioperative contact is limited and recovery often occurs unsupervised.

Therefore, while the haemorrhoidectomy context differs significantly from major abdominal surgery in terms of risk, invasiveness, and hospitalization, it remains a logical extension of ERAS principles. By carefully selecting and adapting high-value interventions—such as opioid-sparing analgesia, early mobilization, and digital follow-up—clinicians can leverage lessons from other surgical domains while tailoring protocols to the unique characteristics of benign anorectal procedures. This critical synthesis supports the development of targeted ERAS pathways that are both evidence-based and operationally feasible in proctology.

## 6 Lessons from major colorectal and emergency surgery

The foundational and most rigorously validated applications of Enhanced Recovery After Surgery (ERAS) protocols originate from elective major colorectal surgery. Within this domain, ERAS pathways have consistently demonstrated significant reductions in postoperative morbidity, accelerated gastrointestinal recovery, shorter hospital stays, and improved patient satisfaction—without increasing complication rates (3, 31, 32). Although initially developed for complex resections, these principles offer highly transferable insights for less invasive operations such as haemorrhoidectomy.

One of the clearest lessons from colorectal ERAS research is the value of standardization through structured, protocolized perioperative care. Evidence from multicenter studies and recent systematic reviews confirms that the systematic application of evidence-based components—such as minimal fasting, preoperative carbohydrate loading, multimodal opioid-sparing analgesia, and early mobilization—leads to reproducible improvements in outcomes (3, 4, 32–34). These interventions target surgical stress responses and are as biologically relevant in minor surgery as they are in major procedures. A focused review by Taplin and O'Connor emphasized that the core physiological goals of ERAS—attenuation of catabolic and inflammatory responses, maintenance of gastrointestinal function, and preservation of muscle mass—apply across surgical intensities (34).

Moreover, colorectal ERAS experience underscores the benefits of minimizing unnecessary interventions, including routine drains, nasogastric tubes, and prolonged immobilization (3, 4, 34). These elements are particularly pertinent to benign anorectal surgery, where pain and delayed return to function are common postoperative barriers. The biological mechanisms addressed by ERAS—such as surgical stress, ileus, and opioid-related complications—are present regardless of surgical complexity.

Critically, the success of ERAS in emergency colorectal surgery further reinforces its adaptability. Traditionally considered unsuitable for ERAS due to unpredictability and high acuity, emergency colorectal operations—such as for obstructive cancer—have shown improved outcomes with modified ERAS pathways. Studies by Shida et al., Shang et al., and others report reductions in postoperative ileus, complication rates, and hospital stay, demonstrating that even in urgent scenarios, structured perioperative management is feasible and beneficial (7, 8, 11, 22, 34).

Another essential takeaway from major colorectal ERAS programs is the necessity of interdisciplinary coordination. Successful implementation depends not only on surgical technique but on collaboration among anesthesiologists, nursing staff, physiotherapists, nutritionists, and care coordinators (16, 21, 31, 32). Even though haemorrhoidectomy is less resource-intensive, adopting a multidisciplinary approach improves consistency in patient education, pain control, discharge planning, and follow-up care.

Patient engagement has emerged as a central determinant of ERAS success. In both oncologic and benign procedures, involving patients in recovery planning, setting realistic expectations, and providing clear postoperative guidance are consistently associated with better compliance and satisfaction (16, 23). These principles are especially relevant in haemorrhoidectomy, where anxiety, fear of pain, and inconsistent home care instructions often lead to unplanned contacts or delays in recovery.

Cost-effectiveness is yet another domain where colorectal ERAS has demonstrated consistent benefit. Economic analyses from large ERAS programs confirm reductions in healthcare expenditures by lowering complication rates, shortening hospital stay, and minimizing resource use (13, 22, 32). Although outpatient haemorrhoid surgery is already cost-efficient, applying ERAS principles could further reduce indirect costs associated with prolonged recovery, analgesic use, and absenteeism—especially in a generally young, employed patient population.

In conclusion, the accumulated experience from ERAS implementation in both elective and emergency colorectal surgery provides a robust theoretical and empirical foundation for its extension to minor proctologic procedures. While procedural differences require tailored adaptation, the overarching goals—improved recovery, reduced variability, optimized patient experience, and resource efficiency—remain consistent and achievable across the surgical spectrum. The main transferable ERAS principles from major colorectal and emergency surgery are summarized in Table 4*.*

**Table 4:** Key ERAS lessons from Major colorectal surgery and their applicability to haemorrhoidectomy.

Domain/ERAS Principle	Key Lessons from Major/Emergency Colorectal Surgery	Implications for Haemorrhoid Surgery
Standardization	Protocolized care improves outcomes across centers (3, 33, 32)	Protocols for pain, feeding, discharge can enhance consistency in day-case care
Early Oral Intake	Reduces ileus, supports metabolic recovery (4, 16)	Promotes bowel function, reduces postoperative constipation
Multimodal Analgesia	Decreases opioid use, enhances recovery (3, 4)	Minimizes side effects (nausea, sedation, urinary retention)
Early Mobilization	Prevents thromboembolism, accelerates function (8, 11)	Enables faster return to daily activity
Avoidance of Drains/Tubes	Reduces complications, shortens stay (4, 34)	Avoid unnecessary interventions (packing, catheterization)
Emergency Surgery Feasibility	Modified ERAS effective even in acute settings (7, 8, 11)	Suggests adaptability to non-complex minor surgery
Multidisciplinary Coordination	Key to successful ERAS implementation (16, 21, 31)	Team-based discharge planning and follow-up valuable even for minor cases
Patient Engagement & Education	Improves satisfaction, reduces anxiety (23, 32)	Essential for outpatient procedures with self-managed recovery
Cost-Effectiveness	Reduces hospital costs and readmissions (22, 23)	Potential to reduce revisits and analgesic-related expenses

Summary of key perioperative principles derived from ERAS implementation in major and emergency colorectal surgery, with direct implications for outpatient haemorrhoidectomy. The table outlines how core ERAS elements—validated in complex surgical contexts—can be effectively adapted to optimize outcomes in minor proctologic procedures.

## 7 Barriers, limitations, and gaps in the literature

Despite the growing interest in applying Enhanced Recovery After Surgery (ERAS) protocols to benign anorectal surgery—particularly haemorrhoidectomy—significant barriers and limitations still characterize the literature. These challenges span methodological shortcomings, heterogeneity in protocol design, limited procedure-specific data, and the paucity of high-quality prospective evidence.

One major gap is the absence of randomized controlled trials (RCTs) exclusively evaluating ERAS protocols in patients undergoing haemorrhoidectomy. The majority of available studies are retrospective or observational, often involving mixed proctologic populations (e.g., fistulotomy, fissure repair, abscess drainage) rather than homogeneous haemorrhoidectomy cohorts (2, 5). While these investigations offer valuable insight into recovery trends, they do not permit robust procedure-specific conclusions regarding efficacy or safety in haemorrhoid surgery *per se*.

Moreover, heterogeneity among studies significantly constrains interpretability. Definitions of ERAS components vary considerably: some pathways explicitly include preoperative patient education and carbohydrate loading, whereas others omit such elements; analgesic regimens are inconsistent, with wide variation in the use of local anesthesia, nerve blocks, or opioid-sparing strategies; postoperative care components such as early feeding, mobilization timing, discharge criteria, and follow-up protocols are implemented variably (1, 3). This variability complicates between-study comparisons and undermines the generalizability of findings.

Another limitation lies in the scarcity of validated, procedure-specific outcome metrics. Most reports rely on generic endpoints such as pain scores, length of stay (LOS), or unplanned consultations, but neglect outcomes highly relevant to haemorrhoidectomy patients—such as time to return to work, early resumption of defecation, disease-specific quality of life, or long-term recurrence/satisfaction rates (4). Without these data, the full clinical value of ERAS in this population remains difficult to quantify and compare across centres.

In addition, few studies address the feasibility of implementing ERAS in diverse healthcare settings, particularly ambulatory day-surgery units or clinics lacking dedicated multidisciplinary ERAS teams. Factors such as workforce training, preoperative education capacity, patient-flow logistics, and infrastructural support may influence pathway effectiveness, yet these implementation variables are seldom examined. Similarly, cost-effectiveness data remain sparse in minor surgery contexts—most economic analyses focus on major inpatient operations (34).

Patient adherence and engagement constitute another under-explored domain. While ERAS is designed to empower patients and improve their recovery experience, studies rarely measure adherence to discharge instructions or investigate how compliance influences outcomes. Psychological and behavioural factors—such as fear of pain, perceived stigma, cultural expectations regarding recovery—may modulate engagement with ERAS components, but these factors are seldom incorporated into study designs (35).

Finally, there is a conspicuous absence of guidelines or consensus statements tailored specifically to ERAS in benign proctologic surgery. Most existing recommendations are extrapolated from colorectal cancer or pelvic surgery guidelines, which may not fully reflect the unique features, logistical constraints, and recovery expectations of outpatient haemorrhoidectomy (36). The lack of a unified, procedure-specific framework limits clinical adoption and results in variability between institutions. These multifactorial limitations—ranging from methodological heterogeneity to the lack of standardized outcome metrics—are consolidated in Table 5 for clarity.

**Table 5:** Key limitations in ERAS literature for haemorrhoid surgery;.

Identified Limitation	Implications for Evidence or Practice	Suggested Solution/Future Direction
Lack of randomized controlled trials (RCTs)	Limits ability to draw causal inferences about ERAS efficacy	Conduct procedure-specific RCTs in haemorrhoidectomy
Heterogeneous ERAS protocol definitions	Prevents comparability and reproducibility across studies	Develop consensus-driven core ERAS components
Limited use of procedure-specific outcomes	Overlooks recovery dimensions relevant to patients	Include PROMs: time to defecation, return to work, QOL
Absence of cost-effectiveness data in outpatient settings	Hinders justification for ERAS implementation in low-resource units	Evaluate downstream cost savings and resource utilization
Poor assessment of patient adherence	Unknown impact of non-compliance on outcomes	Use digital tools to monitor post-discharge behaviors
Minimal consideration of psychological and cultural factors	Recovery experience may vary significantly between populations	Integrate behavioral and psychosocial metrics in trials
Lack of ERAS guidelines for minor proctologic surgery	Leads to variable care and inconsistent adoption	Create and validate ERAS recommendations tailored to haemorrhoidectomy

Summary of key limitations identified in current literature evaluating ERAS protocols in benign anorectal surgery, with emphasis on haemorrhoidectomy. The table outlines the implications for clinical practice and research, along with proposed strategies for improvement.

In summary, while preliminary studies suggest that selected ERAS components may be beneficial in haemorrhoid surgery, the current literature is constrained by methodological limitations, inconsistent implementation, and insufficient focus on procedure-specific outcomes. These gaps underscore the urgent need for prospective, controlled trials, standardized pathway definitions, robust outcome reporting tailored to haemorrhoidectomy, and comprehensive implementation studies to establish a validated ERAS bundle for minor proctologic procedures.

## 8 Future directions and the need for standardized ERAS protocols in proctology

The current trajectory of surgical care continues to favor minimally invasive techniques, ambulatory procedures, and patient-centered recovery models. In this context, haemorrhoidectomy—one of the most frequently performed outpatient operations—represents an ideal candidate for structured implementation of Enhanced Recovery After Surgery (ERAS) protocols. Despite emerging evidence supporting their feasibility and benefits, ERAS pathways for benign proctologic surgery remain underdeveloped, lacking standardization, validation, and widespread clinical adoption. Advancing this field requires coordinated, multidisciplinary efforts to establish procedure-specific, evidence-based ERAS guidelines tailored to the unique features of minor anorectal surgery.

A critical step forward involves developing a core ERAS element set adapted specifically to haemorrhoidectomy. Such a set should be pragmatic, reflecting the realities of ambulatory care, and include simplified fasting protocols, patient education, multimodal analgesia, early feeding and mobilization, and structured discharge planning. While full traditional ERAS protocols designed for major abdominal surgery may not be necessary, a concise “ERAS-lite” model could yield substantial benefits with minimal logistical burden (1, 3, 37). To support the development of such a model, we have included a structured table that outlines the key elements of a proposed ERAS-lite pathway for haemorrhoidectomy. Table 6 presents a phase-based summary of interventions, grouped into preoperative, intraoperative, and postoperative components. Designed to reflect the specific needs and workflow of outpatient proctologic surgery, this visual summary serves as a practical tool to assist clinical teams in conceptualizing and implementing enhanced recovery strategies for minor anorectal procedures.

**Table 6:** Stylized schematic of a proposed “ERAS-lite” pathway for haemorrhoidectomy, outlining key perioperative interventions. The visual grouping reflects phase-specific adaptations relevant to outpatient proctologic surgery.

Preoperative Phase	Intraoperative Phase	Postoperative Phase
Patient education Minimized fasting (clear fluids up to 2 h pre-op) Preemptive analgesia (NSAIDs, paracetamol)	Local or regional anesthesia Short-acting sedation (if needed) Pudendal block/local infiltration No routine catheter or antibiotics	• Early oral intake • Opioid-sparing analgesia • Early mobilization (same day) • Written discharge plan • Follow-up via digital tool or call

Prospective randomized controlled trials are urgently needed to validate such tailored protocols. These studies should assess procedure-specific outcomes including pain trajectories, analgesic consumption, return to normal activity, and patient-reported satisfaction. Furthermore, they should incorporate both objective (e.g., complication rates, unplanned visits) and subjective (e.g., anxiety, recovery confidence, time to social reintegration) endpoints, while integrating longitudinal quality of life measures (2, 4, 38). Importantly, these trials should be stratified by surgical technique (e.g., open vs. stapled haemorrhoidectomy) and anesthesia modality to facilitate comparability.

Beyond clinical validation, the future of ERAS in proctology will depend on advances in implementation science. Effective pathway deployment requires interdisciplinary coordination, staff training, adaptation to local workflow, and integration with institutional logistics. As highlighted by Jones et al., barriers to implementation often lie not in the clinical evidence but in operational execution—including resistance to change, lack of resources, and absence of real-time feedback loops (37). Addressing these factors will be essential for sustained adoption in both high- and low-volume centers. Key strategic priorities for advancing ERAS protocols in haemorrhoid surgery are summarized in Table 7.

**Table 7:** Priority areas for future ERAS development in benign proctologic surgery.

Priority Area	Rationale	Recommended Action
Development of procedure-specific ERAS bundles	Existing protocols are extrapolated from colorectal surgery (1, 3)	Create core ERAS-lite elements tailored to haemorrhoidectomy
High-quality prospective studies	Current data rely on observational or mixed-population studies (2, 38)	Design RCTs measuring pain, function, PROMs, and satisfaction
Implementation science and workflow integration	Barriers exist at institutional level (37)	Develop tools for ERAS adoption in day-surgery workflows
Use of digital health tools	ePROMs and mobile apps improve compliance and recovery (16, 38, 39)	Integrate technology into ERAS pathways for follow-up and symptom tracking
Multidisciplinary consensus guidelines	Lack of unified protocols limits adoption (3, 40)	Formulate ERAS proctology guidelines via expert task forces with patient input
Real-world multicenter collaboration	Lack of comparative data across centers (39)	Establish ERAS registries or collaborative networks for proctologic surgery outcomes

Summary of key priorities for advancing ERAS development in benign proctologic surgery. Each row outlines a focus area, the rationale based on current evidence gaps or challenges, and recommended strategic actions to improve implementation, outcomes, and standardization.

Technology-enabled recovery support offers another powerful tool. Mobile health platforms, digital recovery applications, and ePROMs (electronic patient-reported outcome measures) have demonstrated their ability to support medication adherence, monitor pain progression, detect complications early, and reinforce recovery milestones (16, 38, 40). According to a recent meta-analysis by Lee et al., digital health interventions embedded within ERAS programs were associated with improved patient engagement, reduced readmissions, and higher satisfaction scores in colorectal and pelvic surgery settings (40). Their integration in minor anorectal procedures may allow for continuity of care beyond the operating room and personalized recovery support at scale.

The creation of a dedicated ERAS consensus guideline for benign proctologic surgery remains a pressing priority. A multidisciplinary task force involving surgeons, anesthesiologists, nurses, and patient advocates should lead this initiative. Such a guideline should be stratified by intervention type and anesthesia modality, and include not only clinical recommendations but also practical implementation tools—such as checklists, discharge instruction templates, and patient information leaflets. Inclusion of patient representatives is essential to ensure that recovery pathways align with real-world expectations and preferences (3, 41).

International collaboration and data-sharing networks can further accelerate progress. Establishing a centralized ERAS registry or multicenter collaborative focused on proctologic surgery would enable benchmarking, comparative effectiveness research, and iterative refinement of best practices. Similar networks have propelled ERAS evolution in colorectal, gynecologic, and urologic surgery, and may catalyze comparable progress in outpatient proctology (39).

In conclusion, the future of ERAS in benign proctologic surgery lies in the deliberate evolution from informal adaptation to formal protocolization. By combining rigorous clinical validation, implementation frameworks, digital health integration, and international collaboration, the surgical community can transform perioperative care for one of the most common operations performed today—optimizing recovery, enhancing patient experience, and advancing value-based care in outpatient surgery.

## 9 Conclusions

Although haemorrhoidectomy is a minor surgical procedure, it remains associated with significant postoperative discomfort, unplanned follow-up, and inconsistent recovery experiences. The application of ERAS principles to this setting represents a logical and promising extension of evidence-based perioperative care. Data extrapolated from major colorectal and emergency surgeries, alongside early studies focused on proctologic interventions, suggest that ERAS protocols can safely and effectively improve outcomes even in low-risk outpatient procedures.

Core ERAS elements—such as preoperative education, opioid-sparing analgesia, early mobilization, structured discharge planning, and digital follow-up—are both feasible and impactful when adapted to the unique characteristics of haemorrhoid surgery. While formal evidence remains limited and heterogeneous, the cumulative findings indicate consistent benefits in pain control, patient satisfaction, and reduction in healthcare utilization.

Nevertheless, significant gaps remain in the form of standardized protocols, high-quality randomized studies, and procedure-specific outcome measures. Moving forward, the surgical community must prioritize the development and validation of tailored ERAS pathways for minor proctologic procedures, supported by implementation research and real-world data collection.

By embracing ERAS principles in haemorrhoidectomy, clinicians can deliver more efficient, consistent, and patient-centered care—transforming a traditionally routine intervention into an opportunity for enhanced surgical recovery and modernized perioperative practice.

The successful translation of ERAS principles into diverse surgical specialties reinforces their adaptability beyond colorectal resection. As such, haemorrhoidectomy represents a logical and promising candidate for structured, evidence-based perioperative optimization. Ongoing validation through procedure-specific studies will be essential to confirm these assumptions and refine best practices for benign proctologic surgery.
